# Creating a comprehensive research strategy for cutaneous neurofibromas

**DOI:** 10.1212/WNL.0000000000005789

**Published:** 2018-07-10

**Authors:** Jaishri O. Blakeley, Pierre Wolkenstein, Brigitte C. Widemann, James Lee, Lu Q. Le, Rhonda Jackson, Marigo Stathis, Sharad K. Verma

**Affiliations:** From the Department of Neurology (J.O.B., R.J., M.S., S.K.V.), The Johns Hopkins School of Medicine, The Neurofibromatosis Therapeutic Acceleration Program, Baltimore, MD; Department of Dermatology (P.W.), Paris Est Créteil University, France; Pediatric Oncology Branch (B.C.W.), National Cancer Institute, NIH, Bethesda, MD; Dermavant Sciences (J.L.), Durham, NC; and Department of Dermatology (L.Q.L.), UT Southwestern Medical Center, Dallas, TX.

## Abstract

**Objective:**

Outside of procedural-based methods, there are currently no established medical treatments for cutaneous neurofibroma (cNF), which afflict up to 99% of patients with NF1. Further, adult patients often report cNF are the greatest burden of living with NF1. The Neurofibromatosis Therapeutic Acceleration Program (NTAP) launched a think tank to address core questions to facilitate development of effective therapeutics for cNF in people with NF1.

**Methods:**

Experts (with and without explicit experience with NF1 or cNF) from multiple scientific and medical disciplines, representing the ranks of academia, industry, and government agencies, were invited to become a member of a team addressing a specific subset of questions pertinent to cNF. Teams met monthly to review published and unpublished materials, and created summaries about the material known and unknown that may influence therapeutic development for cNF. Teams prioritized questions and organized supporting data, which was presented to the entire body of experts by each team at a research summit.

**Results:**

Four themes were identified as being relevant to creating a comprehensive research strategy for cNF: (1) establishing definitions of cNF, (2) determining the biology of cNF with respect to tumor initiation, progression, and maintenance, (3) outlining the factors that guide therapies development, and (4) defining core considerations for clinical trials design and optimization for cNF.

**Conclusion:**

Considerations and key questions for each of the thematic areas were identified and provided basis for a request for applications launched by NTAP focused on cNF and are described in the accompanying articles of this supplement.

Cutaneous neurofibromas (cNF) are tumors that bridge neurology and dermatology as they are in the skin and are composed of both dermal and neural tissues. These tumors primarily afflict people with neurofibromatosis type 1 (NF1). NF1 is an autosomal dominant neurogenetic syndrome caused by mutations on chromosome 17q11.2 resulting in the production of abnormal neurofibromin.^1,2^ The protein neurofibromin is highly expressed in the brain, spinal cord, and peripheral nervous system.^3^ In the absence of normal neurofibromin, the Ras-GTPase-activating protein is constitutively activated, contributing to abnormal cell proliferation.^4^

With an incidence of 1/2,500 to 1/3,500, NF1 is among the most common single gene inherited conditions worldwide, affecting all ethnicities, races, and sexes equally.^5^ The clinical criteria for NF1 include: café-a-lait macules, axillary or inguinal freckling, 2 or more cNF or 1 or more plexiform neurofibroma (pNF), presence of an optic nerve glioma, 2 or more Lisch nodules, long-bone abnormalities or sphenoid wing dysplasia, or a confirmed diagnosis in a first-degree relative.^6,7^ Of all of these manifestations, cNFs are among the most common, affecting up to 99% of adults with NF1.^8–11^

A great deal is understood about *NF1* and the cellular and molecular effects of the absence of neurofibromin and there is exciting development of therapies for NF1-associated tumors such as pNF and optic pathway gliomas.^12,13^ However, relatively little is known about cNF. This is partially because cNF are unlikely to cause fatal complications or severe neurologic morbidity. Although cNF are not predisposed to malignant transformation and are rarely associated with physical disability like other *NF1*-driven tumors, they are highly damaging to people with NF1 via disfigurement and dysesthesia. Specifically, in adults with NF1, “perceived disease visibility” is significantly associated with depression, psychosocial distress, quality of life impairment, and negative body experience for attractiveness/self-confidence.^14,15^ Patients report that cNF and their associated symptoms are one of their greatest concerns related to NF1.^16,17^ Hence, cNF represent a major underaddressed health concern for people with NF1. An important limitation to developing a range of effective therapies for cNF is a detailed understanding of the natural history, pathophysiology, and mechanisms underlying cNF development and maintenance.

The Neurofibromatosis Therapeutic Acceleration Program (NTAP), a research enterprise based in the Johns Hopkins School of Medicine focused on therapeutics for NF1-associated peripheral nerve sheath tumors, launched a think tank to address core questions pertaining to the development of effective therapeutics for cNF in people with NF1. To do this, experts (with and without explicit experience with NF1 or cNF) from disciplines across neurology, biology, chemistry, dermatology, surgery, skin cancer, regenerative medicine/tissue repair, wound healing, genetics, and immunology, representing the ranks of academia, industry, and government agencies, were invited to join 1 of 4 teams addressing a specific subset of questions pertinent to cNF. The teams addressed questions related to (1) definition of cNF, (2) tumor initiation, progression, development, (3) therapies development, and (4) clinical trials design, optimization, and development. Teams met monthly remotely to draft core questions, review published and unpublished materials, and create summaries about the material known and unknown that may influence therapeutic development for cNF. Based on these conversations, teams prioritized questions and organized supporting data for presentation to the entire body of experts at a research summit. After these presentations, there were discussions and brainstorming sessions culminating in the teams generating the reports presented in this supplement.

## Defining cutaneous neurofibromas

There is a continuum of benign peripheral nerve sheath tumors in people with NF1. Specifically, neurofibromas in NF1 range from pNF (deep lesions originating in nerve) to subcutaneous neurofibromas (lesions in the hypodermis of skin such that skin can be moved over the tumor) to cNF (currently defined as tumors limited to the skin, epidermis, and dermis, such that they move with the skin).^18–20^ A key limitation for advancing research for these tumors is the great variability in the definitions applied to NF1-associated peripheral lesions preventing efficient therapeutic development in these tumors. The first working group describes the multiple existing published classifications for cNF and discusses the clinical and pathologic features specific to cNF to help lay the framework for a comprehensive nosology for prospective validation.

## Biology of cutaneous neurofibromas

This group defines the current knowledge about the underlying mechanistic, cellular, and genetic factors responsible for the formation of human cNF. They identify possible factors contributing to variable cNF behavior and propose a unified working model for cNF development addressing known and hypothesized aspects of the genetic, cellular, and spatial-temporal factors that may influence development of therapies for cNF.

## Development of therapies for cNF

Current treatments for cNF are limited to procedures addressing individual tumors. Experiences with such approaches are informative for establishing the current standard of care when starting the process of therapeutics development. This group identifies the current treatments and reported outcomes as well as the key factors to consider when creating a therapeutics development strategy for cNF. Specific considerations include the need for agents with favorable toxicity profiles given the likely need for chronic use and defining meaningful benefit in the setting of a complex neurogenetic syndrome.

## Clinical trial design and development paths

The members of this working group review key considerations for clinical trials designed to assess biologic effect, safety and tolerability, and efficacy of therapies for cNF. They formed their assessment from analysis of ongoing natural history studies and the emerging experience with various tools for assessing cNF number, size, and change over time as well as the effect on patients as assessed by patient-reported outcomes. The authors specifically address the endpoints to be considered for cNF that affect trial design and potentially, regulatory review.

## Discussion

The goal of these coordinated efforts is to define the core scientific areas to address in order to develop meaningful therapies for cNF and to inspire new investigation into these unique tumors. For example, related to these efforts, the Cutaneous Neurofibroma Subgroup of the Response Endpoints in Neurofibromatosis and Schwannomatosis (REiNS) International Collaboration launched in order to formally develop and evaluate endpoints for clinical trials focused on cNF (ccrod.cancer.gov/confluence/display/REINS/Cutaneous+Neurofibromas). In addition, to address core scientific questions identified by the authors of this supplement, NTAP released a request for applications (n-tap.org). Through this and related efforts, the diverse scientific community that is committed to improving outcomes for people with *NF1*-associated peripheral nerve sheath tumors will be positioned to make meaningful advances that may affect not only people with NF1, but the broader population of patients with peripheral nerve pathology that affects skin and nerve function.

## Glossary

cNF: cutaneous neurofibromas
NF1: neurofibromatosis type 1
NTAP: Neurofibromatosis Therapeutic Acceleration Program
pNF: plexiform neurofibroma
